# Perceived Food Hypersensitivity Relates to Poor Asthma Control and Quality of Life in Young Non-Atopic Asthmatics

**DOI:** 10.1371/journal.pone.0124675

**Published:** 2015-04-29

**Authors:** Jennifer Johnson, Magnus P. Borres, Lennart Nordvall, Jonas Lidholm, Christer Janson, Kjell Alving, Andrei Malinovschi

**Affiliations:** 1 Department of Women’s and Children’s Health, Uppsala University, Uppsala, Sweden; 2 Thermo Fisher Scientific, Uppsala, Sweden; 3 Department of Medical Sciences, Respiratory Medicine and Allergology, Uppsala University, Uppsala, Sweden; 4 Department of Medical Sciences, Clinical Physiology, Uppsala University, Uppsala, Sweden; University of Athens Medical School, GREECE

## Abstract

**Background:**

The relationship between perceived food hypersensitivity in asthmatics, food allergen sensitization, asthma control and asthma-related quality of life has not been studied.

**Objective:**

Our aim was to study the prevalence of perceived food hypersensitivity in a cohort of young asthmatics, its relation to food allergen sensitization, and any correlation to asthma control and asthma-related quality of life.

**Methods:**

Perceived food hypersensitivity, as well as IgE sensitization to common food allergens, levels of exhaled nitric oxide (FeNO), and blood eosinophil counts (B-Eos) were assessed in 408 subjects (211 women) with asthma, aged (mean ± SEM) 20.4 ± 0.3 years. Subjects filled out the Asthma Control Test (ACT) and the Mini Asthma Quality of Life Questionnaire (Mini-AQLQ). Inflammation was assessed by means of FeNO and B-Eos.

**Results:**

Fifty-three per cent of subjects reported food hypersensitivity. A corresponding food allergen sensitization was found in 68% of these subjects. Non-atopic subjects with perceived food hypersensitivity (n = 31) had lower ACT (19 (15 - 22) vs. 21 (20 - 23), p < 0.001) and Mini-AQLQ -scores (5.3 (4.3 - 6.1) vs. 6.1 (5.5 - 6.5), p < 0.001) than subjects with no food hypersensitivity (n = 190), despite lower levels of FeNO and B-Eos (p < 0.05).

**Conclusions and Clinical Relevance:**

Food hypersensitivity was commonly reported among young asthmatics. In a majority of cases, a corresponding food allergen sensitization was found. A novel and clinically important finding was that non-atopic subjects with perceived food hypersensitivity were characterized by poorer asthma control and asthma-related quality of life.

## Introduction

The prevalence of food hypersensitivity (in our study defined as any adverse reaction upon food intake) is estimated to be between 12% and 20% in adults [1–3]. In a Swedish birth-cohort, food hypersensitivity was reported by parents in 11% of children at 4 years of age [4]. The prevalence of food allergy (in our study defined as IgE-mediated allergic reactions) in the US population is estimated to be almost 10% in adults [5], and 3–6% in children [5, 6]. Twenty-four per cent of asthmatic children included in the School-Inner City Asthma Study had physician-diagnosed food allergy [7]. Food allergy is related to more severe asthma disease, with an increased risk for asthma exacerbations, a higher rate of corticosteroid use, and more frequent hospitalizations [7–14]. The prevalence of perceived food hypersensitivity in an asthma cohort has, to our knowledge, not been investigated, and the relationship between perceived food hypersensitivity, food allergen sensitization, asthma control and asthma-related quality of life has not been studied previously.

Among subjects with asthma, a high prevalence of food allergen sensitization has been reported in children [14] as well as in adults [15]. Food allergen sensitization affects both local and systemic markers of inflammation in asthma [16, 17]. There is a relationship between multiple IgE sensitization and increased prevalence of asthma [18, 19]. In pollen-sensitized individuals, food allergen sensitization increases asthma prevalence and airway inflammation [18].

Our aim was to study the prevalence of perceived food hypersensitivity in an asthma cohort, its relation to food allergen sensitization, and any correlation to manifestations of food hypersensitivity symptoms, asthma control, and asthma-related quality of life.

## Materials and Methods

### Study population

This project was run as a cross-sectional study within the framework of an academy-industry collaboration on Minimally-Invasive Diagnostics for Asthma and allergic diseases (MIDAS) [18, 20]. A total of 408 children and young adults (10–34 years) with physician-diagnosed asthma, recruited from both primary and specialist care in Uppsala, Sweden, and 118 controls with data on perceived food hypersensitivity symptoms and food IgE sensitization were included in the study. All asthma subjects were on daily treatment with inhaled corticosteroids (ICS) and/or oral leukotriene receptor antagonists (LTRA) during at least three months of the year before study entry. The controls were age- and sex-matched controls without asthma or other chronic respiratory diseases, randomly chosen from the population registry.

### Perceived food hypersensitivity symptoms

An allergy nurse conducted interviews, using a structured questionnaire, and each subject was asked to report any history of hypersensitivity reactions to food allergens commonly occurring in Sweden (egg, cow’s milk, fish, wheat, peanut, soy, hazelnut and/or shrimp). Perceived symptoms were grouped according to the organ systems affected: the lower airways (asthma), the upper airways (rhinitis, conjunctivitis), the oral cavity (oral allergy syndrome), the skin (atopic dermatitis, urticaria, angioedema), the gastrointestinal tract (nausea, vomiting, stomach pain, diarrhea), and anaphylaxis (self-reported). Perceived symptoms that did not fit into any group were classified as “other”.

### Measurement of IgE sensitization

Food allergen sensitization was defined as IgE antibody concentration ≥ 0.35 kU_A_/L to egg, cow’s milk, cod fish, wheat, peanut, soy, hazelnut or shrimp. Aeroallergen sensitization was defined as a positive result from the multi-allergen test Phadiatop (Thermo Fisher Scientific, Uppsala, Sweden), a test containing a mix of grass, tree and weed pollen and animal, mite and mold allergens. All IgE analyses were done with ImmunoCAP (Thermo Fisher Scientific). Atopy was defined as any IgE sensitization (≥ 0.35 kU_A_/L) to food and/or aeroallergens.

### Grouping with regard to perceived food hypersensitivity and IgE sensitization

**Fig 1:**
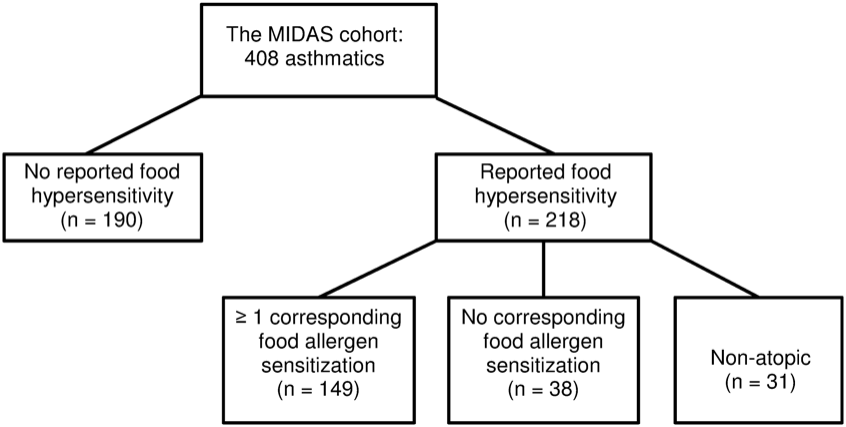
Grouping of asthmatics according to history of perceived food hypersensitivity and IgE sensitization status.

Since we were particularly interested in differences in clinical characteristics in regard to presence or absence of food hypersensitivity symptoms, asthmatics were divided into five groups, depending on perceived history of food reactions and IgE sensitization patterns (Fig 1). The first group consisted of subjects with no history of perceived food hypersensitivity and no detectable IgE sensitization to the food and aeroallergens investigated. The second group consisted of subjects with any detectable sensitization to the food and/or aeroallergens investigated, but without any history of perceived food hypersensitivity. The third group consisted of subjects with a history of perceived food hypersensitivity, and at least one corresponding food allergen sensitization. The fourth group consisted of subjects with a history of perceived food hypersensitivity, but without any corresponding IgE sensitization, and the fifth group consisted of subjects with a history of perceived food hypersensitivity, but without detectable IgE sensitization to the food and aeroallergens investigated (‘non-atopic’).

### Clinical parameters and characteristics

#### Asthma parameters

The fraction of exhaled nitric oxide (FeNO) was measured with a chemiluminescence analyzer (NIOX Flex, Aerocrine AB, Solna, Sweden). Eosinophil blood count (B-Eos) was measured with a routine method (Cell-Dyn Sapphire, Abbott, Illinois, USA). Forced expiratory volume in one second (FEV_1_) was recorded with a MasterScope spirometer (Erich Jaeger, Wurzburg, Germany), and was used as a measure of pulmonary function. The methacholine provocative dose causing a fall of FEV_1_ by 20% (PD_20_) was determined with the Aerosol Provocation System (Viasys Healthcare GmbH, Germany). Measurements were done in accordance with standardized routines and guidelines [21–23], and have been described in detail elsewhere [18, 20].

#### Asthma Control Test and Mini Asthma Quality of Life Questonnaire

Asthmatics were asked to fill out the Asthma Control Test (ACT) and the Mini Asthma Quality of Life Questionnaire (Mini-AQLQ). The ACT consists of five questions, each with a 5-point scale [24]. The score ranges from 5 to 25, with a lower score indicating poorer asthma control. Subjects with an ACT score < 20 were defined as having not well-controlled asthma. The Mini-AQLQ consists of 15 questions, each with a 7-point scale [25]. Both mean total score and for different domains (symptoms, activity limitation, emotional function and environmental stimuli) were calculated. The score ranges from 1 to 7, with a lower score indicating a poorer quality of life.

#### Rhinitis and nocturnal gastro-esophageal reflux

Rhinitis was defined as a positive answer to the question “Do you have any nasal allergies, including hay fever?” [26]. Nocturnal gastro-esophageal reflux (GERD) was defined as heartburn or belching after lying down [27].

#### Body mass index

Height and weight were measured, and body mass index (BMI) was calculated by dividing weight (kg) by the square of height (m). Subjects were subsequently divided into four groups: underweight, normal, overweight and obese. Fixed cut-offs were used for subjects aged ≥ 20 years and percentiles per age and gender for subjects aged < 20 years, as recommended by WHO [28].

#### Asthma medication

The subjects' use of ICS, combination ICS/ long-acting beta-agonists (LABA) and/or LTRA during the last three months was recorded in the interview. The prescribed daily dose of ICS was also collected from the subjects' medical records.

### Statistical analysis

Categorical variables were described using percentages, and differences between groups were studied with the χ^2^ test. Continuous variables that were normally distributed were described using means and standard error of the mean (SEM), and the t-test was used to compare means. If continuous variables had a distribution skewed to the right (e. g. FeNO), a geometric mean with a 95% confidence interval was used for descriptive statistics, and logarithm-transformation was performed before group comparisons. The PD_20_ was described using median and interquartile range, and the Mann-Whitney test was used to compare groups. Single and multiple logistic regression models were used to assess odds ratios for having not well-controlled asthma (ACT< 20). Single and multiple linear regression models were used to study differences in Mini-AQLQ in subjects with food hypersensitivity and different patterns of IgE sensitization, compared with subjects without food hypersensitivity. Age, sex, BMI, FEV_1_, FeNO, total IgE, current dose of ICS, current use of ICS/LABA and current smoking were adjusted for in multiple regression models.

Data were analyzed using the statistics software package Stata (version 12; Stata Corporation, College Station, Texas, USA). P-values ≤ 0.05 were considered significant.

### Ethics

Written informed consent was obtained from each subject, and from parents of subjects below 18 years of age, before inclusion in the study. The study was approved by the Regional Ethics Committee in Uppsala, Sweden (2009/349).

## Results

### Patient characteristics in relation to control subjects

Asthmatic subjects had lower values of FEV_1_, and higher levels of FeNO than controls (Table 1). They also had higher levels of B-Eos, were more often sensitized to both food allergens and aeroallergens, and were more likely to report perceived food hypersensitivity reactions.

**Table 1:** Asthmatics and controls, demographics.

	Asthmatics (n = 408)	Controls (n = 118)
Age (years)^1^	20.4 ± 0.3	20.5 ± 0.6
Female (%)	51	55
FEV_1_ (% pred)	92 ± 0.7***	97 ± 1.3
FeNO (ppb)^2^	15.7 (14.6–16.9) ***	12.4 (11–13.9)
Eosinophils (10^9^/L)^2^	0.18 (0.17–0.2) ***	0.12 (0.10–0.14)
ACT^1^	20.5 ± 0.2	-
Mini-AQLQ-score^1^	5.8 ± 0.05	-
Food allergen sensitization (%)	62***	22
Aeroallergen sensitization (%)	79***	33
Perceived food hypersensitivity (%)	53***	15

^1^Mean ± SEM,

^2^Geometric mean (95% CI),

***p < 0.001

### Prevalence of perceived food hypersensitivity in relation to food allergen sensitization

Food hypersensitivity in asthmatics was most frequently reported to hazelnuts, peanuts, and cow’s milk (Table 2). The food allergen sensitizations most frequently found were against hazelnut, peanut and soy. Concordance between reported food hypersensitivity and food allergen sensitization was most frequently found for reactions to hazelnut, egg and peanut.

**Table 2:** Perceived food hypersensitivity and food allergen sensitization in asthmatics (n = 408), ordered by decreasing prevalence.

Perceived food hypersensitivity (%)	IgE sensitization (%)	Perceived food hypersensitivity and corresponding IgE sensitization (%)
Hazelnut **29**	Hazelnut **54**	Hazelnut **81**
Peanut **22**	Peanut **25**	Egg **62**
Cow's milk **21**	Soy **18**	Peanut **56**
Egg **9**	Wheat **17**	Fish^1^ **55**
Shrimp **8**	Shrimp **15**	Soy **50**
Fish **8**	Egg **12**	Shrimp **47**
Wheat **7**	Cow's milk **12**	Wheat **39**
Soy **6**	Fish^1^ **6**	Cow's milk **20**

The third column shows the percentage of perceived food hypersensitivity where a corresponding IgE sensitization was found.

^1^IgE sensitization to cod fish

### Symptoms of perceived food hypersensitivity in relation to IgE sensitization

Asthmatics with perceived food hypersensitivity were divided into three groups, depending on their IgE sensitization status (Fig 1). Upon food intake, symptoms from the oral cavity and the skin were more prevalent among subjects with at least one corresponding food allergen sensitization than among subjects from the two other groups reporting food hypersensitivity (Table 3). Gastrointestinal symptoms, on the other hand, were less prevalent in subjects with a corresponding food allergen sensitization. Non-atopic subjects with perceived food hypersensitivity had lower prevalence of symptoms from the lower airways associated to food consumption than subjects with a corresponding food allergen sensitization (Table 3).

**Table 3:** Perceived symptoms associated with food consumption (%) among asthmatics.

Perceived food hypersensitivity (n = 218)			
Perceived symptoms (%)	Corresponding IgE sensitization (n = 149)	No corresponding IgE sensitization (n = 38)	Non-atopic (n = 31)
Lower airways	52	34	13***
Upper airways	11	8	-
Oral cavity	77	28***	29***
Skin	42	21*	10***
Gastrointestinal	42	68**	87***
Anaphylaxis	10	5	-
Others	7	8	13

The group with at least one corresponding food allergen sensitization is used as a control group. All p-values refer to comparisons between this group and the others.

*p < 0.05,

**p < 0.01,

***p < 0.001

### Asthma characteristics in relation to perceived food hypersensitivity and IgE sensitization

#### Univariate analyses

Subjects with perceived food hypersensitivity and at least one corresponding food allergen sensitization had higher levels of FeNO, B-Eos and total IgE than subjects without any perceived food hypersensitivity (Table 4). The former group also reported more episodes of wheeze and asthma attacks in the past 12 months (Table 5). For atopic subjects with perceived food hypersensitivity without any corresponding food allergen sensitization, no such differences were seen. Non-atopic subjects with perceived food hypersensitivity had lower levels of FeNO, B-Eos and total IgE than those without perceived food hypersensitivity. Non-atopic subjects without perceived food hypersensitivity had lower levels of FeNO, B-Eos and total IgE when compared to atopic subjects within the same group (data not shown).

**Table 4:** Asthmatics with and without perceived food hypersensitivity, divided by IgE sensitization status, demographics.

		Perceived food hypersensitivity (n = 218)		
	No perceived food hypersensitivity (n = 190)	≥ 1 corresponding IgE sensitization (n = 149)	No corresponding IgE sensitization (n = 38)	Non-atopic (n = 31)
Age (years)^1^	20.2 ± 0.5	20.6 ± 0.6	21.5 ± 1.2	19.3 ± 1
Female (%)	49	51	58	65
Normal BMI (%)	62	67	66	55
Overweight (%)	20	16	21	24
Obese (%)	16	12	11	21
Underweight (%)	2	5	2	-
Current smoking (%)	4.2	3.4	7.7	3.2
Rhinitis (%)	66	89***	90***	45*
GERD (%)	12	15	18	19
FEV_1_ (%)^1^	92 ± 1	90.9 ± 1.2	95.4 ± 2.2	93.6 ± 2.3
FeNO (ppb)^2^	14.2 (12.7–15.8)	19.4 (17.2–21.9)***	16.7 (12.3–22.7)	9.8 (8.3–11.6)**
Methacholine challenge test (PD_20_)^3^	0.3 (0.1–2.9)	0.2 (0.05–1.2)*	0.3 (0.1–1.9)	0.8 (0.2–1.8)
Eosinophils (10^9^/L)^2^	0.16 (0.15–0.19)	0.23 (0.19–0.26)***	0.18 (0.13–0.24)	0.12 (0.09–0.15)*
Total IgE (kU_A_/L)^2^	95 (75–120)	325 (265–399)***	162 (106–246)	25 (16–38)***
Inhaled corticosteroids (μg/day)^3^	389 (358–423)	407 (366–452)	416 (348–496)	414 (301–569)
Continuous use of single ICS last 3 months (%)	40	38	28	48
Continuous use of ICS/LABA last 3 months (%)	42	46	56	32
Continuous use of LTRA last 3 months (%)	15	21	23	19

All p-values refer to comparisons between the group without perceived food hypersensitivity and the others.

^1^Mean ± SEM,

^2^Geometric mean (95% CI),

^3^Median (IQR),

*p < 0.05,

**p < 0.01,

***p < 0.001

**Table 5:** Asthmatics with and without perceived food hypersensitivity, divided by IgE sensitization status.

		Perceived food hypersensitivity (n = 218)		
Asthma symptoms (%)	No perceived food hypersensitivity (n = 190)	≥ 1 corresponding IgE sensitization (n = 149)	No corresponding IgE sensitization (n = 38)	Non-atopic (n = 31)
Wheeze?	68	78*	74	68
Awoken by chest tightness?	31	38	34	39
Shortness of breath during rest?	23	31	24	32
Shortness of breath after exercise?	70	77	71	87*
Awoken by shortness of breath?	14	17	5	16
Asthma attack?	57	68*	74	68
Emergency visit due to asthma attack/exacerbation?	15	17	24	23

Asthma symptoms in past 12 months. All p-values refer to comparisons between the group without perceived food hypersensitivity and the others.

*p < 0.05

**Fig 2:**
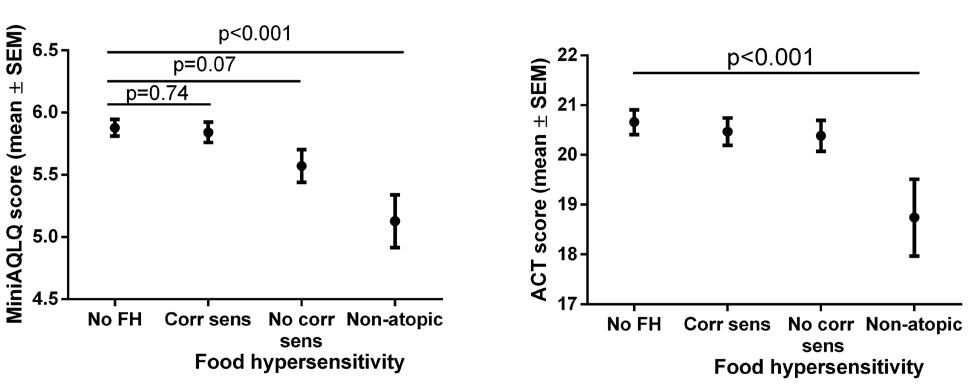
Mini-AQLQ- and ACT-scores in relation to perceived food hypersensitivity and IgE sensitization status.

**Fig 3:**
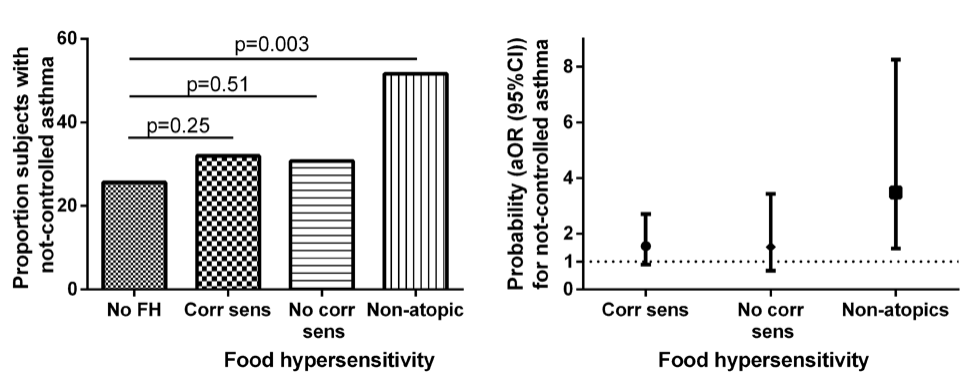
Proportion of subjects with not well-controlled asthma (Panel A) and adjusted odds ratios (age, sex, BMI, FEV_1_, levels of FeNO, total IgE, current dose of ICS, current use of ICS/LABA, and current smoking) (Panel B) for not well-controlled asthma in relation to perceived food hypersensitivity and IgE sensitization.

Non-atopic subjects with perceived food hypersensitivity scored lower on the ACT (Fig 2, S1 Table), and had higher prevalence of not well-controlled asthma (Fig 3A) than subjects without perceived food hypersensitivity. Non-atopic subjects with perceived food hypersensitivity scored lower on overall Mini-AQLQ (Fig 2, S1 Table), as well as on all four domains of the Mini-AQLQ (data not shown), than subjects without perceived food hypersensitivity. Among those without perceived food hypersensitivity, no difference was found between non-atopic and atopic subjects in ACT or Mini-AQLQ scores (S2 Table). Non-atopic subjects with food hypersensitivity had lower ACT and Mini-AQLQ scores as compared to atopic subjects with food hypersensitivity. Furthermore, a trend towards lower ACT score and a lower Mini-AQLQ score were found in non-atopic subjects with food hypersensitivity *vs* atopic subjects with food hypersensitivity, while no differences in ACT or Mini-AQLQ scores were found in similar analyses in atopic subjects comparing subjects with and without food hypersensitivity (S2 Table).

#### Multivariate analyses

The probability of having not well-controlled asthma was 3.1 (1.4–6.7) (OR (95% CI)) times higher in the non-atopic group with perceived food hypersensitivity than in the group with no perceived food hypersensitivity. This relation was consistent (3.5 (1.5–8.3)) after adjusting for age, sex, BMI, FEV_1_, FeNO, total IgE, current dose of ICS, current use of ICS/LABA and current smoking (Fig 3B). The results regarding lower Mini-AQLQ—scores in non-atopic subjects with perceived food hypersensitivity, as compared with those without perceived food hypersensitivity, were also consistent after adjustments for the same factors as above: the mean differences being 0.77 (0.39–1.15) for overall Mini-AQLQ, 0.96 (0.46–1.3) for symptoms domain, 0.74 (0.35–1.13) for activity limitation, 0.66 (0.12–1.20) for emotional function, and 0.63 (0.14–1.13) for environmental stimuli. After adjustments, atopic subjects without any corresponding food allergen sensitization had a lower environmental domain score, mean difference 0.72 (0.27–1.16), than those without perceived food hypersensitivity.

## Discussion

Approximately half of the investigated young asthmatics reported food hypersensitivity, and a corresponding food allergen sensitization was found in a majority of them. There were large variations for different foods in concordance between perceived food hypersensitivity and food allergen sensitization. Differences in perceived symptoms upon food intake were found between subjects with and without corresponding food allergen sensitization. A novel and clinically important finding of the present study was that non-atopic subjects with perceived food hypersensitivity, despite having lower levels of FeNO and B-Eos, had poorer asthma control, were more likely to have not well-controlled asthma, and had lower asthma-related quality of life than those without perceived food hypersensitivity. Subjects with perceived food hypersensitivity and at least one corresponding food allergen sensitization had similar levels of asthma control and asthma-related quality of life as those without perceived food hypersensitivity, despite having higher levels of the above mentioned inflammation markers.

Both food allergen sensitization and perceived food hypersensitivity were more common among asthmatic subjects than controls. The prevalence of perceived food hypersensitivity among control subjects in our study (15%) corresponds well to prevalence rates found in earlier population-based surveys [1–3], while the prevalence rate for asthmatics was much higher (53%). In a pediatric asthma cohort, physician-diagnosed food allergy, which included only IgE-mediated allergic reactions, was present in 24% of asthmatic children [6].

A large proportion of subjects with perceived food hypersensitivity had corresponding IgE sensitization, although the percentage of concordance between perceived symptoms and detectable IgE antibodies differed between foods. The highest concordances were found for hazelnut, egg and peanut. The high rate of IgE sensitization to hazelnut found in our study can to a large extent be explained by cross-reactivity to birch pollen [29], since the study took place in a region with frequent birch-pollen sensitization. This is also true for IgE sensitization to peanut, where cross-reactivity occur between Ara h 8 and Cor a 1. There is an association between IgE sensitization and allergy to egg early in life, and development of respiratory allergic disease some years later [30]. In a Swedish birth-cohort study, self-reported symptoms caused by peanut were closely associated to IgE sensitization to peanut among 4-year-old children [4]. Sensitization to hazelnut was not investigated in this birth-cohort study.

Symptoms from the lower airways, the oral cavity, and the skin were more frequently reported among subjects with at least one corresponding food allergen sensitization than among non-atopic subjects. The opposite was true for symptoms from the gastrointestinal tract, which were more frequently reported among non-atopic subjects. Non-IgE-mediated food reactions seem to primarily affect the gastrointestinal tract [31]. Since many of the perceived food hypersensitivity reactions in this study were reactions to cow’s milk, we could not exclude that these reactions were to some extent due to lactose intolerance. The prevalence of lactose intolerance is, however, lower in Sweden than in other parts of the world [32].

To our knowledge, the relationship between perceived food hypersensitivity in non-atopic asthmatics and impaired asthma control and asthma-related quality of life, has not been reported before. The relationship between perceived food hypersensitivity, asthma control and asthma-related quality of life has not been well studied, although the presence of food allergy has been linked to increasing asthma severity [7–14]. American children with confirmed food allergy exhibit health-related quality of life (HRQL) scores below the national average [33]. In a Swedish birth-cohort study, 9-year old children with reported food hypersensitivity were found to score lower on some HRQL subscales, as compared to children with other allergic diseases (asthma, eczema or rhinitis) [34].

Subjects with perceived food hypersensitivity and corresponding food allergen sensitization had higher levels of local and systemic markers of Th2-driven inflammation. This corresponds to previous results from the same patient material, where IgE sensitization to food allergens was recently found to affect both local and systemic markers of inflammation in asthma [18]. A discrepancy between increasing levels of inflammation markers and subjectively perceived asthma control was found. Discrepancies between patient perceptions and objective ratings of asthma severity have previously been described [35–37], and may in part be explained by differences in personality [37–39]. In our cohort, overall levels of both local and systemic inflammation markers were relatively low, indicating a well-treated asthma population.

The strength of the present study is the inclusion of asthma patients from both primary and specialist care. Inclusion of age- and sex-matched controls has allowed comparison of prevalence of food hypersensitivity and food allergen sensitization between asthmatics and the general population. A further strength is the large study material, which has allowed subgrouping and comparisons between groups. We acknowledge the limitation of perceived food hypersensitivity, as differences exist between perceived symptoms and objectively confirmed food hypersensitivity [40–42]. The main objective of this study was, however, to focus on patient perceptions of food hypersensitivity, asthma control and asthma-related quality of life. Another limitation of perceived food hypersensitivity may be the reporting of gastrointestinal symptoms. These symptoms can be unspecific and related to other causes than food hypersensitivity, such as irritable bowel syndrome, indigestion or gastrointestinal infections. Any of these gastrointestinal disorders may lead to decreased ACT- score or quality of life. However, this is a less likely explanation as few individuals reported these conditions in the questionnaires or interviews. A third limitation of the present study is that subjects were asked to report if they had *ever* experienced symptoms associated with food consumption. This could include episodes in early childhood. The discrepancy between perceived food hypersensitivity and food allergen sensitization may thus in part be explained by development of tolerance to food allergens [43]. Unfortunately, we also lack information on whether subjects with perceived food hypersensitivity are currently consuming the suspected food stuff or not.

In conclusion, approximately half of the investigated young asthmatics in our study reported food hypersensitivity, and a majority of these had corresponding food allergen sensitization. Clinical manifestations of perceived food hypersensitivity differed with the presence or absence of corresponding food allergen sensitization. This is the first report of a relationship between perceived food hypersensitivity, impaired asthma control and asthma-related quality of life in non-atopic asthmatics. This was despite lower levels of inflammation, as assessed by levels of FeNO and B-Eos. Food hypersensitivity in non-atopic asthma should be mapped further regarding other inflammatory mechanisms, and asthma management of these patients should be reassessed as they appear to have low levels of Th2-related inflammation.

## Supporting Information

S1 Table Asthma control (ACT) and asthma-related quality of life (Mini-AQLQ, total score and score for different domains), presented as median (range), in relation to perceived food hypersensitivity and IgE sensitization status. (DOCX)

S2 Table ACT and Mini-AQLQ scores (mean values ± SEM) for atopic asthmatics and non-atopic asthmatics, with and without perceived food hypersensitivity. (DOCX)
