# Supplementary material for: Perceived Food Hypersensitivity Relates to Poor Asthma Control and Quality of Life in Young Non-Atopic Asthmatics
Source: PLoS One. 2015 Apr 29;10(4):e0124675. doi: 10.1371/journal.pone.0124675 (PMC4414584; doi:10.1371/journal.pone.0124675)
Supplement: S1 Table — (DOCX) [file pone.0124675.s001.docx]

**Supplementary Table 1.** Asthma control (ACT) and asthma-related quality of life (mAQLQ total score and score for different domains), presented as median (range), in relation to perceived food hypersensitivity and IgE sensitization status.

|  | **No perceived food hypersensitivity**  (n = 190) | **≥ 1** **corresponding IgE sensitization**  (n=149) | **No corresponding IgE sensitization**  (n = 38) | **Non-atopic**  (n = 31) |
| --- | --- | --- | --- | --- |
| ACT (median (range)) | 21 (10, 25) | 21 (9, 25) | 21 (16, 25) | 19 (10, 25) |
| mAQLQ total score  ((median (range)) | 6.1 (2.1, 7) | 6.2 (1.9, 7) | 5.7 (3.5, 6.9) | 5.3 (2.9, 6.9) |
| mAQLQ symptom score  (median (range)) | 5.8 (2.2, 7) | 6 (1.8, 7) | 5.4 (3, 6.8) | 4.8 (2.2, 6.8) |
| mAQLQ activity score  (median (range)) | 6.5 (2.3, 7) | 6.3 (1.8, 7) | 6 (4.3, 7) | 5.5 (1.8, 7) |
| mAQLQ emotional score (median (range)) | 6 (1, 7) | 6 (1.3, 7) | 5.7 (2, 7) | 5 (2.7, 7) |
| mAQLQ environmental score (median (range)) | 6.3 (1.3, 7) | 6.3 (2, 7) | 5.7 (3, 7) | 5.7 (2, 7) |
