# Supplementary material for: Perceived Food Hypersensitivity Relates to Poor Asthma Control and Quality of Life in Young Non-Atopic Asthmatics
Source: PLoS One. 2015 Apr 29;10(4):e0124675. doi: 10.1371/journal.pone.0124675 (PMC4414584; doi:10.1371/journal.pone.0124675)
Supplement: S2 Table — (DOCX) [file pone.0124675.s002.docx]

**Supplementary Table 2.** ACT and Mini-AQLQ scores (mean values ± SD) for atopic asthmatics and non-atopic asthmatics, with and without perceived food hypersensitivity.

| **ACT** | Atopy | | |
| --- | --- | --- | --- |
| Perceived food hypersensitivity | + | p value | - |
| + | 20.6 ± 0.2  (n = 187) | 0.06 | 18.7 ± 0.8  (n = 31) |
| p value | 0.51 |  | 0.007 |
| - | 20.9 ± 0.3  (n = 140) | 0.54 | 20.4 ± 0.5  (n = 50) |

| **Mini-AQLQ** | Atopy | | |
| --- | --- | --- | --- |
| Perceived food hypersensitivity | + | p value | - |
| + | 5.8 ± 0.1  (n = 176) | 0.01 | 5.1 ± 0.2  (n = 31) |
| p value | 0.18 |  | <0.001 |
| - | 5.9 ± 0.1  (n = 136) | 0.22 | 5.8 ± 0.1  (n = 50) |
